# Pancreatic Diffuse Large B-cell Lymphoma in the US Population

**DOI:** 10.7759/cureus.39862

**Published:** 2023-06-02

**Authors:** Asad Ullah, Kue T Lee, Kali Malham, Abdul Qahar Khan Yasinzai, Bisma Tareen, Dara Lopes, Agha Wali, Luis Velasquez Zarate, Abdul Waheed, Maya Wiest, Resham Hakim, Marjan Khan, Bina Asif, Nikhil Patel, Sahar Hakim, Kaleemullah Kakar, Saleh Heneidi, Nabin R Karki, Feroze Sidhwa

**Affiliations:** 1 Pathology, Vanderbilt University Medical Center, Nashville, USA; 2 Otolaryngology, Augusta University Medical College of Georgia, Augusta, USA; 3 Gastroenterology, Augusta University Medical College of Georgia, Augusta, USA; 4 Surgery, Bolan Medical College, Quetta, PAK; 5 Internal Medicine, Bolan Medical College, Quetta, PAK; 6 Surgery, San Joaquin General Hospital, French Camp, USA; 7 Pathology, Augusta University Medical College of Georgia, Augusta, USA; 8 Internal Medicine, San Joaquin General Hospital, French Camp, USA; 9 Internal Medicine, Marshfield Medical Center, Marshfield, USA; 10 Medicine, Bannu Medical College, Bannu, PAK; 11 Cardiology, San Joaquin General Hospital, French Camp, USA; 12 Pathology, Cedars-Sinai Medical Center, Los Angeles, USA; 13 Oncology, Mitchell Cancer Institute, University of South Alabama, Mobile, USA; 14 General Surgery/Trauma and Critical Care, San Joaquin General Hospital, French Camp, USA

**Keywords:** multivariable analysis, chemotherapy, seer, diffuse large b-cell lymphoma, pancreatic lymphomas

## Abstract

Background: Pancreatic lymphomas (PLs) represent <2% of all lymphomas and <0.5% of all pancreatic neoplasms. An accurate histologic diagnosis of PL is needed to predict prognosis and adequately treat the patient. This study aims to investigate the demographic, clinical, and pathological factors affecting the prognosis and survival of pancreatic diffuse large B-cell lymphoma (DLBCL).

Methods: Demographic and clinical data from 493 cases of DLBCL of the pancreas were identified between 2000 and 2018 using the Surveillance, Epidemiology, and End Results (SEER) database.

Results: The most common age group was between the ages of 70 and 79 years (27.0%). While 44% of cases involved distant sites (a proxy for secondary pancreatic DLBCL), regional and localized involvement was seen in 33%, with the most common cause of death being a primary pancreatic DLBCL. Most patients (71%) received only chemotherapy (systemic therapy). The overall five-year observed survival was 46% (95% CI, 43.5-48.3). The one-year and five-year survival with chemotherapy only was 68% (95% CI, 65.3-70.3) and 48% (95% CI, 44.7-50.5), respectively. The one-year and five-year survival with surgery and chemotherapy was 96% (95% CI, 91.3-99.9) and 80% (95% CI, 71.4-89.2), respectively. Surgery with chemotherapy (HR: 0.397 (95% CI, 0.197-0.803), p = 0.010) were both positive predictors in survival prognosis. Multivariable analysis identified age >55 years (HR: 2.475 (95% CI, 1.770-3.461), p < 0.001), distant stage (HR: 6.894 (95% CI, 4.121-11.535), p < 0.001), and undergoing no surgery (HR: 2.610 (95% CI, 1.307-5.215), p = 0.007) as negative predictors for survival.

Conclusion: PLs are rare malignant pancreatic neoplasms with DLBCL being the most common histological subtype. An accurate and timely diagnosis of pancreatic DLBCL is necessary to implement effective treatments and reduce mortality. Systemic therapy (chemotherapy) with or without surgical therapy improved survival. Increased age and regional and distant spread negatively impacted survival.

## Introduction

Pancreatic lymphomas (PLs) are rare pancreatic neoplasms representing <0.5% of all pancreatic tumors and <2% of lymphomas [1]. While primary PL originates in the pancreas and may involve surrounding lymph nodes, secondary PL also involves non-regional lymph nodes or other extra-nodal sites. Secondary PL is more common than primary PL, although the most common histological subtype of both primary and secondary PL is diffuse large B-cell lymphoma (DLBCL) [2]. Lymphomas are categorized as either Hodgkin or non-Hodgkin lymphomas (NHL) [3]. While Hodgkin lymphomas rarely disseminate to extra-lymphatic organs, NHL commonly affects extra-nodal organs, with the gastrointestinal tract being the most common site (30-40%) [4,5]. Extra-nodal involvement of the pancreas by NHL is an extremely rare occurrence observed in only 0.6% of NHL originating from the pancreas (primary PL) [6]. Distinguishing pancreatic DLBCL from more common pancreatic lesions such as pancreatic adenocarcinoma is critical, as the five-year survival rate for pancreatic adenocarcinoma is 5%, while the survival rate for pancreatic DLBCL is 26-66% [7,8]. Therefore, an accurate diagnosis can make a considerable difference in prognosis and treatment strategy. Endoscopic ultrasound-guided fine-needle aspiration (EUS-FNA) is the gold standard in the diagnosis of solid and cystic pancreatic neoplasms [9]. EUS-FNA may offer an accurate diagnosis with minimal safety risk to the patient while remaining cost-effective in comparison to more invasive procedures such as laparotomy or laparoscopy [10].

The objective of this study is to investigate the demographic, clinical, and pathological factors affecting the prognosis and survival of pancreatic DLBCL.

## Materials and methods

Using the Surveillance, Epidemiology, and End Results (SEER) database, patients with the diagnosis of DLBCL of the pancreas during 2000-2018 were obtained. This database contains around 28% of the United States population using 18 different registries, which are the Alaska Native, Arizona Indians, Cherokee Nation, Connecticut, Detroit, Georgia Center for Cancer, Greater Bay Area Cancer, Greater California, Hawaii, Iowa, Kentucky, Louisiana, New Jersey, Seattle-Puget Sound, and Utah Tumor Registry from SEER software (https://seer.cancer.gov/seerstat/; accessed: March 5, 2023). The data collected from these registries include men and women, and for the race, it includes White, Black, Asian, Native American, and Alaska natives. The data collected from hospitals, clinics, and hospice care are reported to the registries of the SEER database.

These data were then exported to SPSS version 20.2 (IBM Corporation, Armonk, NY). Demographic factors that were extracted include age, sex, and race. Clinical factors that were extracted were tumor stage, treatment characteristics, cause of death, overall survival, and survival time. These variables were collected as they were available in the SEER database. Cases with unknown histology or status based on "autopsy only" were excluded. Endpoints that were examined in this study included overall survival and mortality at one, two, three, four, and five years.

This study was performed using the IBM SPSS v28.0.0.0 (190) software to perform multivariate analysis on various factors affecting survival and to create Kaplan-Meier survival curves. Analysis of variance (ANOVA) was performed to identify statistically significant independent variables for the Cox regression model; for this step, significance was set at a p-value of 0.25. Multivariate Cox regression analysis was used to calculate hazard ratios for the identified independent factors affecting survival. Statistical significance was set at a p-value of <0.05.

## Results

Demographic data

Of the patients in this study, the median age was found to be 68 years. The most common age group was between the ages of 70 and 79 years (27%, 133), followed by 60 and 69 years (25%, 125). The youngest age in this cohort was 14 years old (Table 1).

**Table 1:** Demographic factors and tumor characteristics of DLBCL in the US population DLBCL: diffuse large B-cell lymphoma.

Variable (N = 493)		Frequency (%)
Age (years)	1-19	3 (0.6%)
	20-29	3 (0.6%)
	30-39	16 (3.2%)
	40-49	36 (7.3%)
	50-59	88 (17.8%)
	60-69	125 (25.4%)
	70-79	133 (27.0%)
	≥80	89 (18.1%)
Sex	Male	293 (59.4%)
	Female	200 (40.6%)
Race	White	420 (85.2%)
	Black	32 (6.5%)
	Asian or Pacific Islander	32 (7.5%)
	Native American	3 (0.6%)
	Unknown	1 (0.2%)

Regarding sex, the majority of cases in this cohort were male (59%, 293), and the remaining 41% (200) were female. Regarding race, the majority of cases were White (85%, 420), followed by Black (6%, 32), and Asian or Pacific Islanders (7%, 32) (Table 1).

Tumor and treatment characteristics

The tumor stage of 9% (45) of cases was unknown. When known, most cases (44%, 199) were distant, followed by regional (33%, 149) and localized (22%, 100) stages. In SEER, the localized stage is defined as cases that were confined to the organ of origin of primary pancreatic DLBCL. In this study, we consider pancreatic DLBCLs with distant stages to be proxies for secondary pancreatic DLBCL and those with regional and local stages to be proxies for primary pancreatic DLBCL.

In this cohort, 288 (58.4%) patients were deceased. The cause of death was due to the pancreas as the primary site in 9% (25) of cases. The cause of death was due to other causes in 91% (263) of cases. Other sites included Hodgkin lymphoma, non-Hodgkin lymphoma, breast, ovary, stomach, esophagus, lung, and bronchus. Other causes of death in this cohort include myeloid leukemia, atherosclerosis, cerebrovascular diseases, chronic liver disease and cirrhosis, chronic obstructive pulmonary disease, congenital anomalies, diabetes mellitus, heart diseases, benign or unknown behavior neoplasm, melanoma, nonmelanoma skin cancer, miscellaneous malignant cancer, nephritis, nephrotic syndrome, other infectious and parasitic diseases, suicide and self-afflicted injury, and other causes of death (Table 2).

**Table 2:** Cause of death of patients with DLBCL in the US population DLBCL: diffuse large B-cell lymphoma.

Primary site & cause of death (N = 493)		
Status	Alive	205 (41.6%)
	Dead	288 (58.4%)
Cause of death (N = 288)		
Cause	Primary site	185 (64.2%)
	Other sites	103 (35.8%)

Metastasis and treatment in DLBCL

**Figure 1:**
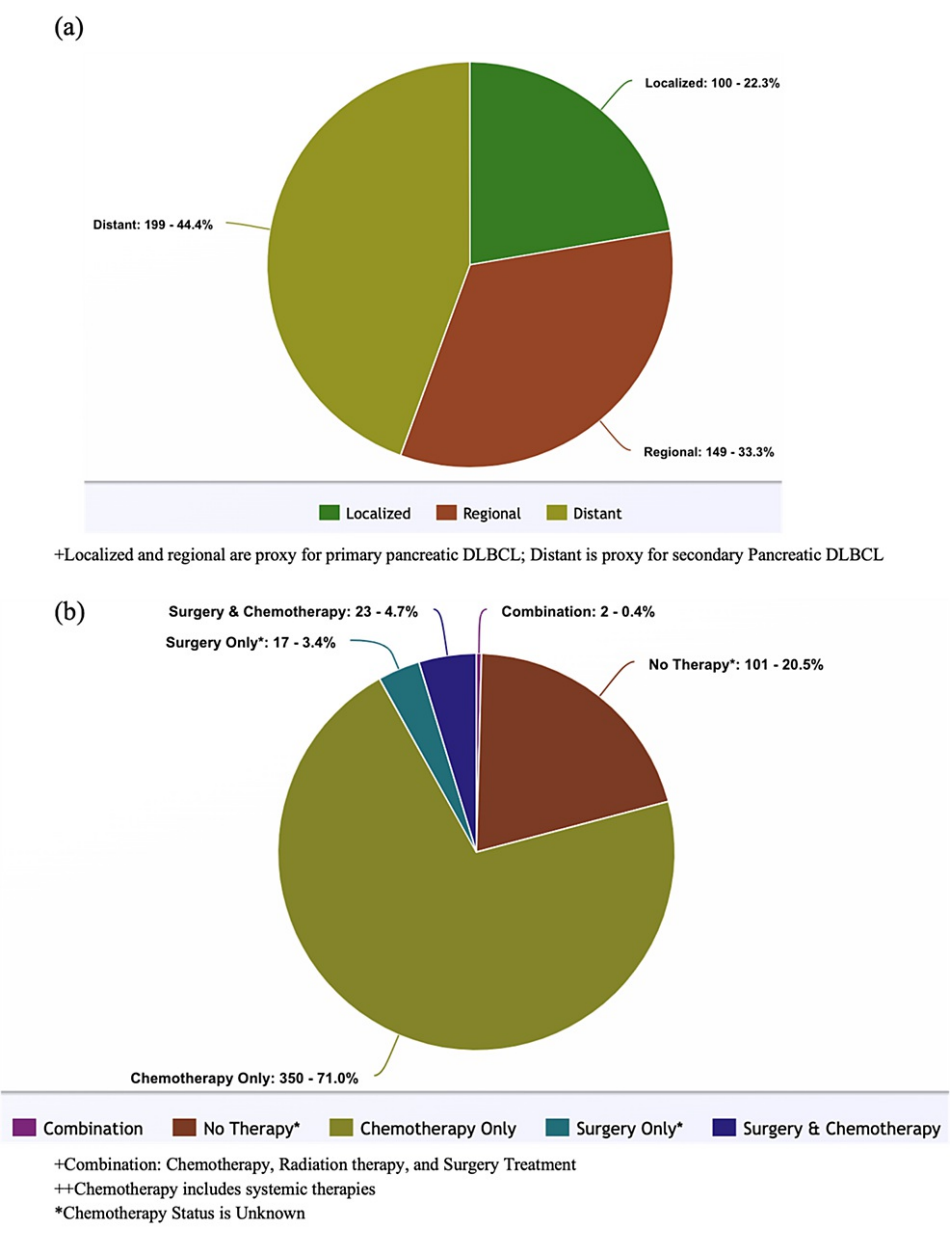
Pie chart of (A) tumor stage and (B) treatment characteristics in patients with DLBCL in the US population DLBCL: diffuse large B-cell lymphoma.

A regional stage is defined as cancer spread only by direct extension to adjacent organs, tissues, and/or lymph nodes of primary pancreatic DLBCL. Both localized and regional stages are proxies for primary pancreatic DLBCL. The distant stage is characterized by cancer spreading beyond the adjacent organs and metastasizing to distant lymph nodes or tissues. The distant stage is a proxy for secondary DLBCL. In the entire cohort, 22% (100) were organ-confined (pancreas), 33% (149) had a regional disease, and 44% (199) had a distant disease (Figure 1A).

Of all cases, 24% (118) had an unknown systemic therapy (labeled as chemotherapy in data description, tables, and figures henceforth) status. Most cases in this cohort underwent chemotherapy only (71%, 350), 3% (17) of cases had surgery only, 5% (25) of cases underwent both surgery and chemotherapy, 0.4% (2) of cases underwent combination therapy (surgery, radiation, and chemotherapy), and 21% (101) of cases did not undergo any treatment (Figure 1B).

Outcomes and survival analysis

**Figure 2:**
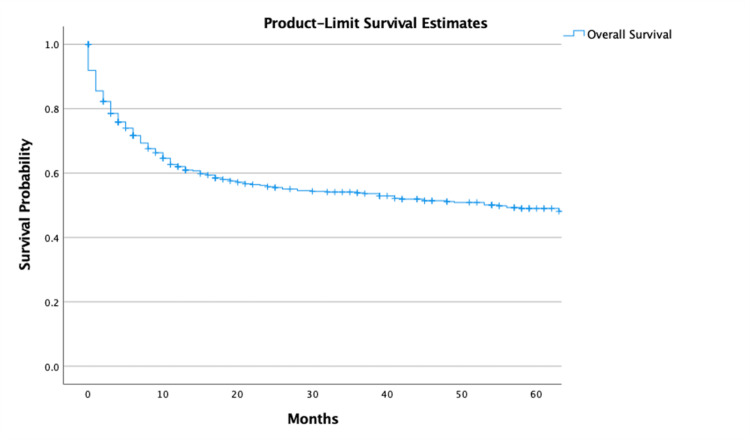
Overall survival in patients with DLBCL in the US population DLBCL: diffuse large B-cell lymphoma.

**Figure 3:**
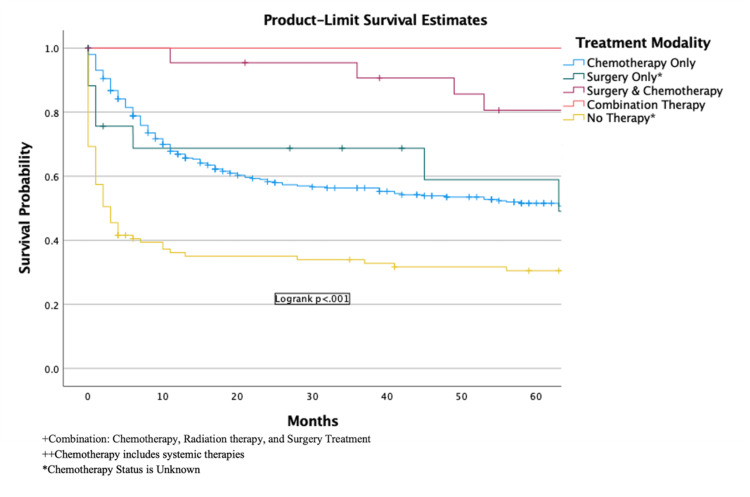
Survival of different treatment modalities in patients with DLBCL in the US population DLBCL: diffuse large B-cell lymphoma.

The overall five-year observed survival was 46% (95% CI, 43.5-48.3) (Figure 2). The one-year and five-year survival with chemotherapy only was 68% (95% CI, 65.3-70.3) and 48% (95% CI, 44.7-50.5), respectively. The one-year and five-year survival with surgery and chemotherapy was 96% (95% CI, 91.3-99.9) and 80% (95% CI, 71.4-89.2), respectively. Survival analysis of surgery only, combination therapy, and other therapies not included in this analysis could not be performed due to a limited number of cases. There was a statistically significant difference (p < 0.001) among all therapies (Figure 3).

Survival analysis by race and sex

**Figure 4:**
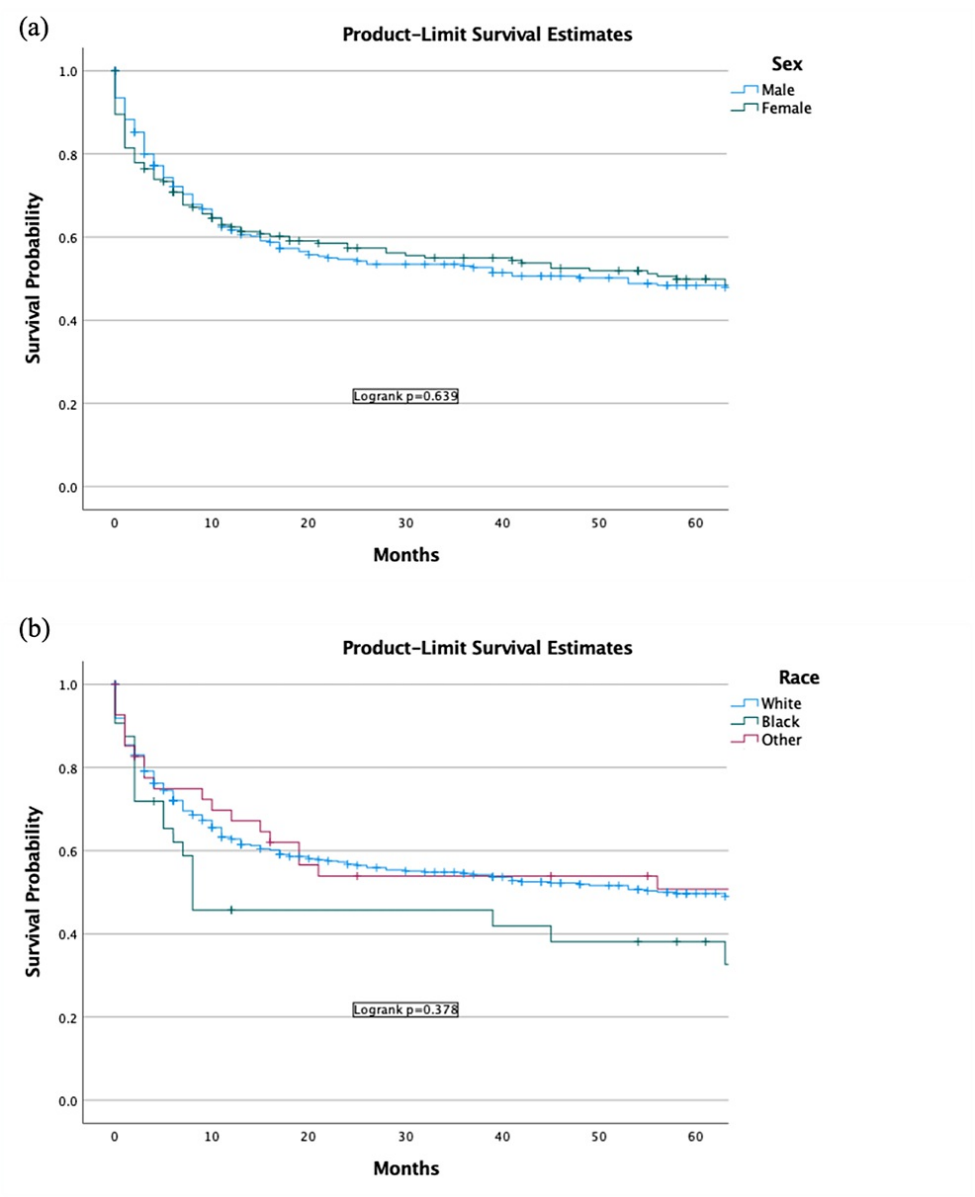
Survival by (A) race and (B) sex in patients with DLBCL in the US population DLBCL: diffuse large B-cell lymphoma.

The one-year survival for White Americans was 57% (95% CI, 54.6-59.6), and the five-year survival rate was 46% (95% CI, 43.7-48.9). For Black Americans, the one-year survival was 46% (95% CI, 37.1-54.9), and the five-year survival rate was 33% (95% CI, 24.1-42.3). For other races, the one-year survival was 54% (95% CI, 46.2-62.2), and the five-year survival rate was 51% (95% CI 43.0-59.2). Race was not a predictor of survival (Figure 4A).

The survival in males at one year was 55% (95% CI, 51.8-57.8), and the five-year survival rate was 44% (95% CI, 41.2-47.4). The survival rate for females at one year was 58% (95% CI, 54.5-61.7), and the five-year survival rate was 48% (95% CI, 44.4-51.8). Sex was not a predictor of survival (Figure 4B).

Survival analysis by age and extent of disease

**Figure 5:**
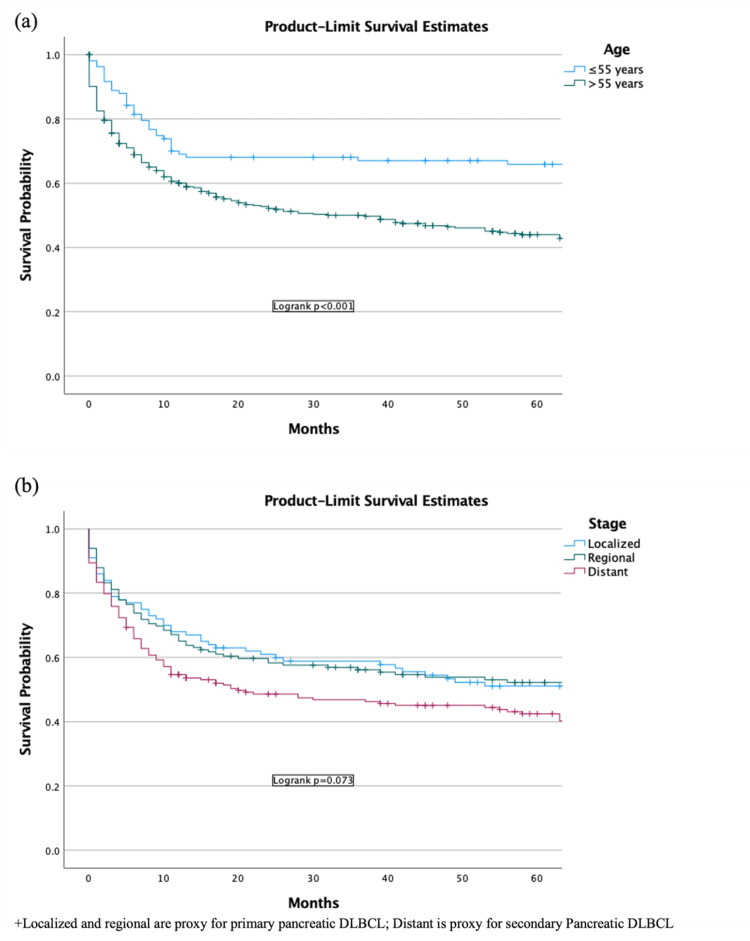
Survival by (A) age and (B) tumor stage in patients with DLBCL in the US population DLBCL: diffuse large B-cell lymphoma.

Age was split into two cohorts: <55 and ≥55 years. Increased age was considered a poor indicator of survival (p < 0.001) (Figure 5A). There was no significant survival difference noted for localized and regional disease. However, distant diseases were associated with the overall lowest survival (p = 0.073) (Figure 5B).

Univariate and multivariable analysis

Univariate analysis revealed that not undergoing any therapy was a negative predictor in survival prognosis (HR: 12.126 (95% CI, 1.634-2.765), p < 0.001), while chemotherapy only (HR: 0.711 (95% CI, 0.555-0.911), p < 0.007) and surgery with chemotherapy (HR: 0.397 (95% CI, 0.197-0.803), p = 0.010) were both positive predictors of survival prognosis (Table 3). The univariate analysis also indicated that increased age was a negative predictor (HR: 1.504 (95% CI, 1.118-2.023), p = 0.007) of survival. Univariate analysis revealed that regional stage (HR: 1.779 (95% CI, 1.094-2.891), p = 0.025) and distant stage (HR: 5.484 (95% CI, 3.369-8.926), p < 0.001) are considered negative predictors for survival.

**Table 3:** Univariate and multivariate analysis of independent factors influencing mortality of DLBCL in the US population * Associated with worse outcomes. ** Associated with better outcomes. + Chemotherapy includes systemic therapies.

­Variables	Univariate		Multivariate	
	Hazard ratio (95% CI)	p-value	Hazard ratio (95% CI)	p-value
Age ≥ 55 years	2.466 (1.764-3.448)	<0.001*	2.475 (1.770-3.461)	<0.001*
Chemotherapy only	0.711 (0.555-0.911)	<0.007**	1.312 (0.671-2.567)	0.427
Surgery + chemotherapy	0.397 (0.197-0.803)	0.010**	0.581 (0.224-1.508)	0.265
No therapy	2.126 (1.634-2.765)	<0.001*	2.610 (1.307-5.215)	0.007*

Multivariable analysis through Cox survival regression analysis identified age >55 years (HR: 2.475 (95% CI, 1.770-3.461), p < 0.001), distant stage (HR: 6.894 (95% CI, 4.121-11.535), p < 0.001), and undergoing no surgery (HR: 2.610 (95% CI, 1.307-5.215), p = 0.007) as negative predictors for survival (Table 3).

## Discussion

PLs are rare, extra-nodal presentations of NHL representing less than 2% of all lymphomas. The most common type of PL is DLBCL among both primary and secondary PLs [7]. In this study, we analyzed a large database of patients with PL to evaluate the demographic, clinical, and pathologic factors that influence survival. Overall, primary (regional and local stages combined) was more common than secondary (distant spread) pancreatic DLBCL. In pancreatic DLBCL, secondary involvement and regional involvement (in primary pancreatic DLBCL) were negative predictors of survival. Systemic therapy (chemotherapy) with or without surgery improved survival, and most patients received systemic therapy only. While previous literature has shown that secondary pancreatic DLBCL is reported more commonly than primary pancreatic DLBCL [2], our study reports the contrary. This may be due to the non-inclusion of systemic DLBCL with pancreatic involvement in our “pancreatic DLBCL with distant spread” category, as well as suboptimal staging. In our opinion, while the secondary pancreatic DLBCL category is likely more accurate, the primary pancreatic DLBCL category may underestimate the number of cases, as positron emission tomography-computed tomography (PET-CT) may not have been utilized for the staging of these pancreas-based lymphomas.

Facchinelli et al. demonstrated that the median age of patients with primary pancreatic lymphoma (PPL) was 53 years, and 64% of the patients were male. The most common presentation was abdominal pain with a median tumor size of 60 mm. In 63% of patients with PPL, the tumor was localized in the pancreatic head. Most lymphomas of the pancreas were DLBCL (53%), although, in patients less than 18 years, most were Burkitt lymphoma (52%). Of the patients, 20% had disease progression and metastasis to the CNS [11]. A population-based study by Gupta et al. demonstrated the demographics and survival analysis of extra-nodal DLBCL lymphoma. The results of their study show that 34% of all DLBCL are extra-nodal, and this tumor occurs more commonly in males (54%) and non-Hispanic Whites (71%). In extra-nodal disease, most were diagnosed above 60 years of age and presented with early staged (69%) disease. Higher mortality was observed in White patients and in patients with late presentation and a history of second malignancies. In comparison to intestinal tract DLBCL, a disease involving CNS, pancreas, and the respiratory system was associated with worse survival [12].

Our findings add to the literature that PL is most common in those aged 70-79 years. Similarly, advanced age was a negative predictor of survival. PL was most common in White Americans, although neither age nor sex impacted survival rates.

Treatment and prognosis of PL depend on the stage and type, with high levels of lactate dehydrogenase (LDH) and beta-2 microglobulin indicating a poorer prognosis [7,13]. The Revised International Prognostic Index (R-IPI) is the predominant tool used to predict the prognosis of aggressive NHL. The R-IPI prognostic factors are as followed: age > 60 years, stage III/IV disease, elevated LDH, Eastern Cooperative Oncology Group performance status (ECOG PS) of 2 and above, and >1 extra-nodal site of disease. Patient risk groups are assigned based on the number of prognostic factors and are divided into “very good” (0 risk factors), “good” (one to two risk factors), and “poor” (three to five risk factors) [14]. Our findings that increased age, regional spread, and distant spread negatively impact survival are consistent with and support these guidelines.

Chemotherapy is the mainstay of treatment in patients with PL, and it demonstrates a better prognosis when compared to the treatment of pancreatic adenocarcinoma [7,8]. The first-line chemotherapy regimen for PL consists of cyclophosphamide, vincristine, doxorubicin, and prednisone, with the addition of rituximab if CD20 positive (R-CHOP) [15]. Second-line treatment in patients with refractory DLBCL consists of one of two chemotherapy regimens, each with comparable efficacy: rituximab, ifosfamide, carboplatin, and etoposide (R-ICE) or rituximab, dexamethasone, high-dose cytarabine, and cisplatin (R-DHAP) [16]. Recent studies have suggested that the use of lenalidomide-rituximab chemotherapy in refractory cases of DLBCL has fewer adverse events than other commonly used second-line therapies [17]. The use of combined chemoradiotherapy has also been reported in the treatment of PL, with one study demonstrating a mean survival of 26 months in patients receiving combined chemoradiotherapy, compared to 13 months in patients receiving chemotherapy alone [13]. Surgical resection of PL has remained a therapeutic challenge due to the large tumor size and high risk of postoperative pancreatic fistula [18]. However, previous studies have reported an increase in five-year survival rate in PL patients treated with a combination of surgery and adjuvant chemotherapy when compared to chemotherapy alone [19,20]. Our findings that chemotherapy and chemotherapy with surgery improve one-year and five-year survival are consistent with these data.

Limitations

Limitations of this study include limitations common to any retrospective study, including the availability of data for analysis. For many patients, the systemic therapy status was not known. The sample size for those receiving surgery alone and radiation therapies did not allow for analysis of these therapies. Finally, comorbid conditions impacting survival could not be analyzed in this study.

## Conclusions

PL is a rare malignant neoplasm of the pancreas that comprises less than 1% of all pancreatic tumors, and DLBCL is the most common histological subtype of both primary and secondary PLs. In this study, we found that increased age was associated with a poorer prognosis in pancreatic DLBCL. We found that systemic therapy with or without surgery significantly improved survival. Although rare, accurate and timely diagnosis of pancreatic DLBCL is necessary to implement effective treatments and reduce mortality. Overall, primary pancreatic DLBCL was more common than secondary pancreatic DLBCL. Involvement of peripancreatic structures and nodes in primary pancreatic DLBCL and secondary involvement in systemic DLBCL were associated with a worse prognosis. We aim that the results of this study will help clinicians to assess long-term prognosis and patient counseling about the overall demographics and clinicopathological patterns of this rare entity. We also anticipate that clinicians and pathologists consider primary pancreatic DLBCL in differentials while assessing specimens on fine needle aspiration cytology.
